# Xp11.2 Translocation Renal Cell Carcinoma: A Rare Renal Cell Carcinoma

**DOI:** 10.7759/cureus.37072

**Published:** 2023-04-03

**Authors:** Pranjal Kalita, Biswajit Dey, Animesh Saurabh, Sheikh F Chishti

**Affiliations:** 1 Pathology, North Eastern Indira Gandhi Regional Institute of Health and Medical Sciences (NEIGRIHMS), Shillong, IND

**Keywords:** immunohistochemistry(ihc), clear cell renal cell carcinoma, tfe3, xp11.2, translocation-associated renal cell carcinoma

## Abstract

Translocation-associated renal cell carcinoma (TRCC) is a group of under-recognized malignant renal neoplasms owing to the unavailability of ancillary diagnostic tools and considering the fact that these tumors may histomorphologically mimic a heterogeneous group of neoplasms ranging from benign to malignant ones. Xp11.2 translocation-associated renal cell carcinoma is a disease of the young with a relatively less known prognosis owing to the rarity of such reported neoplasms. The histological appearance of bulbous tumor cells with abundant, vacuolated cytoplasm and the presence of psammomatoid bodies are clues to the diagnosis but are not entirely specific. The immunohistochemistry (IHC) finding of transcription factor E3 (TFE3) positivity is an important pointer, but the demonstration of Xp11.2 translocation by fluorescence in situ hybridization (FISH) serves as the confirmatory test. In our case report, we highlight the fact that a combined approach involving light microscopy, immunohistochemistry, and fluorescence in situ hybridization is the key to its diagnosis.

## Introduction

Translocation-associated renal cell carcinoma (TRCC) is a rare variant of renal cell carcinoma (RCC) that is slowly getting recognized considering the accessibility and advances in ancillary diagnostic modalities [1]. The first case of Xp11.2 TRCC was reported by Dijkhuizen et al. in the year 1995; however, it was only in the year 2004 that the World Health Organization (WHO) categorized this group of TRCC as a separate entity in its classification of urinary system tumors [2,3]. Xp11.2 translocation RCC represents a rare and independent variant of RCC, characterized by translocations involving the transcription factor E3 (TFE3) gene located on chromosome Xp11.2 with numerous other genes [4]. This rare variant of RCC is more frequently diagnosed in pediatric patients and rarely in adults, with women having a slightly higher incidence than men [4]. The prevalence of Xp 11.2 TRCC is 20-40% among pediatric renal tumors, whereas in the adult population, a low prevalence of only 1-1.6% is noted [5]. Exact data on the prognosis of this rare carcinoma are yet to be known; however, cases diagnosed in children with isolated lymph node metastasis have a comparatively favorable outcome [1]. Adults usually present with widespread metastasis at presentation and have a far worse prognosis [1]. RCC with Xp11.2 translocation is rare in India and is an under-recognized entity.

We report a case of Xp 11.2 translocation RCC in a middle-aged female to highlight the utility of the ancillary technique in the accurate diagnosis of such carcinomas, which can histologically mimic several other RCCs, and also to highlight that TRCC, an infrequent renal carcinoma in adult populations, shows a predominance among the female population, as highlighted in the medical literature.

## Case presentation

A 50-year-old middle-aged female presented to the outpatient department (OPD) with complaints of pain in the left loin for one year and swelling over the left abdominal region for three months. In elaborating on the past history, the patient had no history of hypertension or diabetes mellitus, nor did the patient have any metabolic ailments. There was no family history of cancer-related deaths in her family.

On examination, the patient was conscious, oriented, and afebrile with a blood pressure (BP) of 106/62 mm Hg and a pulse rate (PR) of 108/min. Abdominal examination revealed a 20 cm × 15 cm firm swelling over the abdomen involving the left hypochondriac, lumbar, umbilical, and epigastric regions. Examination of the central nervous system, cardiovascular system, and respiratory system was unremarkable.

Laboratory findings revealed hemoglobin of 8.2 g/dL, a total leucocyte count of 6.0 × 103/L, serum urea, and creatinine levels of 30 and 1.5 mg/dL, respectively. The results of the liver function test and the electrolyte levels of the patient at presentation were within the normal range. Viral markers for hepatitis B surface antigen (HBsAg), anti-hepatitis C virus (anti-HCV), and human immunodeficiency virus (HIV) were negative.

Radiological evaluation of the patient by contrast-enhanced computed tomography (CECT) showed a heterogeneous large left renal mass with left renal vein thrombus and multiple secondaries in the lung, which were presumed to be nodules consistent with metastases.

The patient was subjected to a cytoreductive left-open nephrectomy under general anesthesia. Operative findings revealed a left renal mass measuring 20 cm × 20 cm × 10 cm arising from the upper pole of the left kidney with a dilated left renal vein. The lower pole of the kidney was free of tumors.

A specimen labeled left renal mass weighing 1246 g was received. No cortico-medullary junction demarcation was noted. The outer surface is gray-white, with areas of hemorrhage and an attached, easily strippable capsule. The cut section showed a variegated appearance along with multiple cystic, solid, and hemorrhagic areas. A grossly normal-appearing kidney measuring 7 cm × 2.5 cm was noted. The mass was replacing the renal sinus.

**Figure 1:**
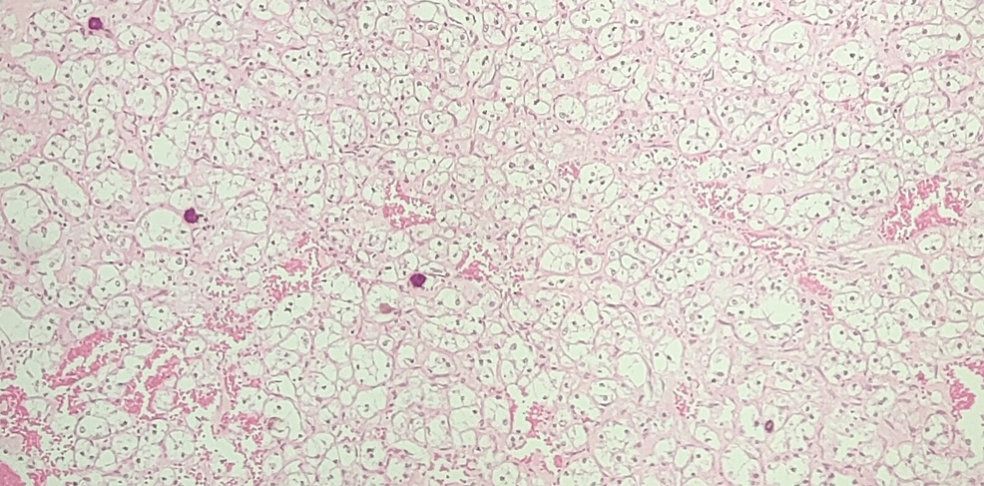
Low power magnification showing tumor consists of cells arranged in solid sheets, alveolar, and papillary architecture along with characteristic psammoma bodies (H&E, 100×)

Microscopy examination of hematoxylin and eosin (H&E) slides showed bulbous tumor cells arranged in solid, papillary, and alveolar architecture with abundant clear to eosinophilic cytoplasm, vesicular chromatin, and prominent nucleoli. Psammoma bodies and mitotic figures were also noted (Figure 1).

**Figure 2:**
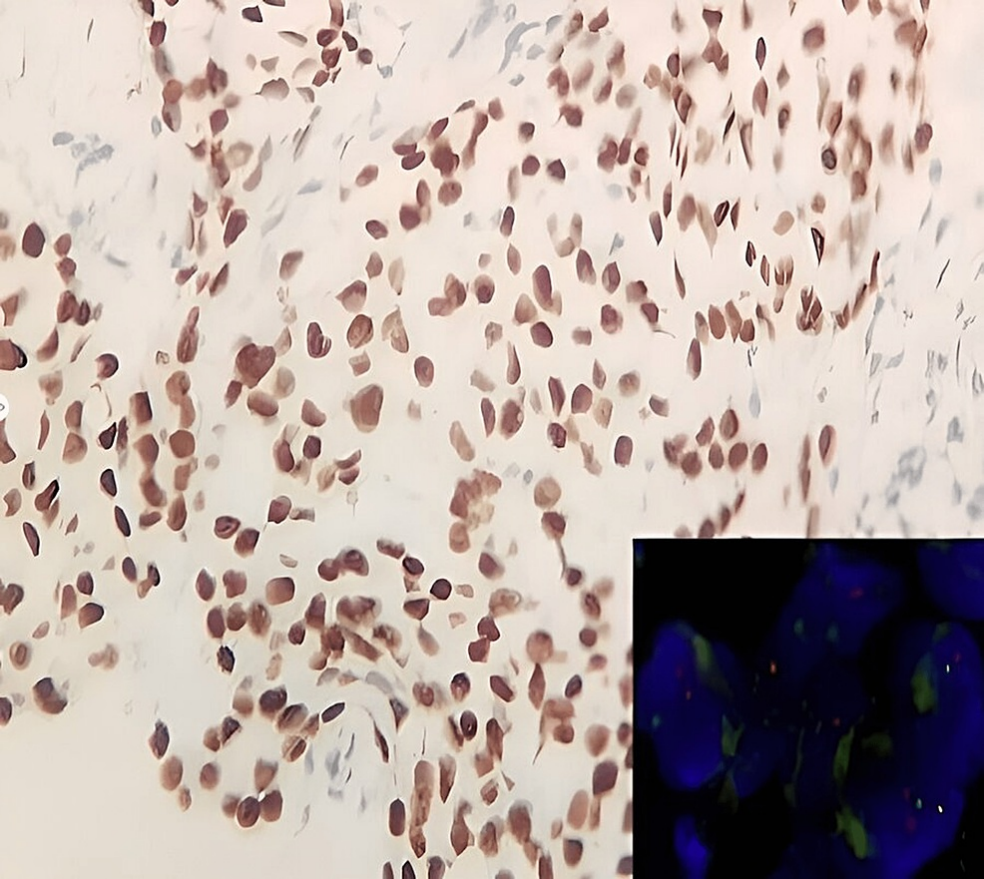
Immunohistochemistry showing tumor cells with nuclear positivity for TFE3 (IHC, 200×); inset showing dual color, break-apart fluorescence in situ hybridization showing Xp11.2 translocation

Immunohistochemistry (IHC) showed TFE3 and epithelial membrane antigen (EMA) positivity, whereas the IHC stains for cytokeratin (CK-7), alpha-methyl acyl-CoA racemase (AMACR), S100, and vimentin were negative (Figure 2).

Dual-color, break-apart fluorescence in situ hybridization (FISH) was utilized to identify the split signals for TFE3 translocation. Translocation of Xp11.2 was confirmed as the FISH sample showed 86% translocation for TFE3 (Figure 2 inset).

Considering the histomorphology, immunohistochemistry, and fluorescence in situ hybridization findings, a diagnosis of Xp11.2 translocation renal cell carcinoma was considered. The International Society of Urological Pathology (ISUP) grade was 2. The renal sinus showed infiltration by tumor cells.

The patient was advised to follow up one month after her discharge, but she did not turn up for follow-up examinations, and on further investigation by the authors, the family members revealed that the patient expired 1.5 months after her surgery.

## Discussion

Traditionally, RCCs have been classified based on predominant cytoplasmic features (e.g., clear cell and chromophobe RCC), architectural features (e.g., papillary RCC), anatomical location (e.g., collecting duct RCC), specific renal disease background (e.g., acquired cystic disease-associated RCC), distinctive molecular alterations (e.g., MIT family translocation RCC), and familial predisposition syndromes (e.g., hereditary leiomyomatosis and RCC syndrome-associated RCC) [6]. The new WHO 2022 classification of RCC introduced molecularly defined RCC categories, which include mainly SMARCB1-deficient medullary RCC, anaplastic lymphoma kinase (ALK)-rearranged RCC, elongin C (ELOC)-mutated RCC, transcription factor EB (TFEB)-altered RCC, and TFE3-rearranged RCC [6]. These molecularly identified RCC subtypes may require more individualized therapy approaches in addition to having prognostic value [6].

Xp11.2 translocated renal cell carcinoma is a comparatively newly recognized malignant renal cell neoplasm involving chromosome Xp11.2, located in TFE3 transcription factor gene fusion with various other genes [7]. Partner genes for TFE3 include proline-rich mitotic checkpoint control factor (PRCC), alveolar soft part sarcoma (ASPSCR), protein-associated splicing factor (PSF), non-POU domain-containing octamer-binding (NonO or p54nrb), and clathrin heavy chain (CLTC) [7]. As this tumor harbors a TFE3 gene translocation, which fuses with one of several other partner genes, it is also known as TFE3-rearranged renal cell carcinoma.

Xp11.2 translocated RCC shows a wide variation in its prevalence, ranging from 20-40% among the pediatric RCC, whereas among the adult population, it is a rare neoplasm, accounting for approximately 1-1.6% of all adult RCC [8]. Adult Xp11.2 translocation RCC was more frequently diagnosed in females and was associated with a larger tumor size and a higher propensity for extracapsular spread, metastasis, advanced disease, and a poorer prognosis [9]. Case reports on this rarer renal neoplasm from India are sparse considering the rarity of such neoplasms and the absence of fluorescence in situ hybridization facilities in various diagnostic laboratories [1,10]. Grossly, Xp11.2 TRCC showed a variegated appearance with areas of hemorrhage and necrosis and a predominantly tan-yellow cut surface. Microscopy of TRCC was non-specific, and histomorphology alone may be confused with various renal cell carcinomas. In our case, a series battery of IHC markers was utilized to aid us in ruling out various renal neoplasms that may mimic TRCC, as summarized in Table 1.

**Table 1:** Immunohistochemistry panel required to rule out mimickers of translocation-associated renal cell carcinoma TFE3: transcription factor E3; CK7: cytokeratin 7; AMACR: alpha-methylacyl-CoA racemase

IHC markers	Interpretation
Vimentin: negative;TFE3: positive	Rules out clear cell renal cell carcinoma
CK7: negative; TFE3: positive	Rules out chromophobe renal cell carcinoma
CK7: negative, vimentin: negative; AMACR: negative	Rules out papillary renal cell carcinoma
S100: negative; TFE3: negative	Rules out oncocytoma

Light microscopy findings along with IHC marker findings helped in differentiating this entity from its mimickers. However, for a complete workup, FISH studies were carried out, which confirmed our diagnosis by demonstrating the characteristic Xp11.2 translocation.

The malignant potential of TRCC is roughly equivalent to clear cell renal cell carcinoma (ccRCC) and exceeds that of papillary renal cell carcinoma [11]. Surgery is the first line of treatment in patients without metastasis, whereas in patients presenting with widespread metastasis, the role of targeted and immunotherapy is being considered [1, 11]. In patients with metastasis, the median survival period for the patients treated with sunitinib versus a group of patients treated by immune therapy was 8 and 2 months, respectively, in a study done by Malouf et al. [12]. Although the study by Malouf et al. showed interesting findings, the study consisted of a very small study population [12]. Considering the unavailability of targeted or immunotherapy primarily due to financial constraints and the unavailability of such drugs, along with no clear-cut data suggesting the potential benefits, our patient was managed conservatively post cytoreductive surgery, and she expired 1.5 months after her surgery.

Our case of TRCC in a middle-aged female with widespread metastasis highlights the fact that TRCC is a disease not primarily limited to the pediatric age group and also points out that TRCC in adults is associated with a higher rate of metastasis as compared to that in children. Routine utilization of ancillary diagnostic modalities will help pathologists and oncologists pick up this under-recognized variant of renal cell carcinoma.

## Conclusions

Xp11.2 translocation RCC is an under-recognized entity that is combined with histomorphology, immunohistochemistry, and FISH studies to aid in the diagnosis of this rare RCC. Adult Xp11.2 translocation RCC is a disease frequently associated with adult females and presents with higher metastasis and a poorer prognosis in spite of recent advances in targeted as well as immunotherapy.
